# Leaf litter mixtures alter decomposition rate, nutrient retention, and bacterial community composition in a temperate forest

**DOI:** 10.48130/FR-2023-0022

**Published:** 2023-09-27

**Authors:** Kun Li, Ying Lu, Qing-Wei Wang, Ruiqiang Ni, Rongchu Han, Chuanrong Li, Caihong Zhang, Weixing Shen, Qi Yao, Yueyin Gao, Sergio de-Miguel

**Affiliations:** 1 Mountain Tai Forest Ecosystem Research Station of State Forestry Administration/Key Laboratory of State Forestry Administration for Silviculture of the Lower Yellow River, Tai'an 271018, Shandong, PR China;; 2 CAS Key Laboratory of Forest Ecology and Management, Institute of Applied Ecology Chinese Academy of Sciences, Shenyang 110016, PR China;; 3 Research Center for Forest Carbon Neutrality Engineering of Shandong Higher Education Institutions/Key Laboratory of Ecological Protection and Security Control of the Lower Yellow River of Shandong Higher Education Institutions, Tai’an 271018, Shandong, PR China ;; 4 Mount Tai Scenic Spot Management Committee, Tai'an 271000, Shandong, PR China; 5 State-owned Guangping Forest Farm, Chiping District, Liaocheng 252100, Shandong, PR China; 6 Department of Crop and Forest Sciences, University of Lleida, Av. Alcalde Rovira Roure 191, E-25198 Lleida, Spain; 7 Joint Research Unit CTFC–AGROTECNIO-CERCA, E-25280 Solsona, Spain

**Keywords:** Bacterial community, Mixture effect, Nutrient cycling, Non-additive effect

## Abstract

Litter decomposition is a key step in global biogeochemical cycling. In forest ecosystems, litter from different tree spec1ies often decompose together. Although species diversity is widely acknowledged to accelerate decomposition through the regulation of nutrient transfer between litter and decomposer communities, the underlying mechanism remains unclear. To explore the association between the bacterial community and mixed-litter chemical transformation, we conducted a one-year litter mixing decomposition experiment using leaf litter from four dominant tree species in Mount Tai (Eastern China), *Robinia pseudoacacia*, *Quercus acutissima*, *Pinus tabulaeformis*, and *Pinus densiflora*. Our results showed that: 1) Mass loss of leaf litter mixtures was significantly faster than that of leaf litter monocultures, except for *R. pseudoacacia*. Litter mixtures without *R. pseudoacacia* showed non-additive synergistic effects, whereas litter mixtures with *R. pseudoacacia* exerted additive effects; 2) Litter species in the absence of *R. pseudoacacia* significantly decreased the nutrient retention rates of litter mixtures compared to those of monocultures; 3) Litter mixtures with or without *R. pseudoacacia* showing additive and non-additive effects in monocultures had a distinct bacterial community structure; 4) Bacterial community structure was also modified by initial litter traits; carbon (C), nitrogen (N), and phosphorus (P) concentrations in monocultures; N/P and C/N ratios of mixtures with *R. pseudoacacia*; and the lignin/N ratio of mixtures without *R. pseudoacacia*. Overall, these findings indicate that tree species diversity controls decomposition and nutrient cycling, implying that an appropriate species community composition is beneficial to maintaining forest ecosystems.

## Introduction

Decomposition of litter is crucial for terrestrial biogeochemical cycles, and it is widely acknowledged that the litter decomposition rate is regulated by climate, litter quality, and decomposer communities^[1−4]^. Litter quality is the primary factor governing the rates at which litter decomposes across different biomes globally^[5,6]^. The decomposition rates vary greatly depending on the substrates, which can simultaneously cause the rapid decline in easily accessible nutrients and the accumulation of more resistant compounds^[7,8]^. Microorganisms are important components of biogeochemical cycling and ecosystem functioning^[9]^. In ecosystems, microorganisms are the primary decomposers of plant litter, and they are more important than detritivores in litter mixtures^[10,11]^. Although the litter decomposition rate and its regulatory factors have been intensely investigated, most of our current knowledge is derived from litter monoculture studies^[12,13]^. Thus, most findings are not necessarily applicable to natural ecosystems, where different types of litter decompose together.

Much attention has been given to species diversity and its effects on litter decomposition and terrestrial nutrient cycles^[14,15]^ because litter type, species richness, and litter–species interactions significantly drive litter decomposition^[16,17]^. Studies have been reported that decomposition rates increase with greater litter diversity^[18,19]^, whereas no relationship or an opposite trend was observed by others^[20,21]^. This discrepancy may be due to interactions among litters comprised of different tree species, which can affect decomposer communities^[8,19,22]^. Compared with monospecific litter, mixed litters may alter the physical and chemical properties of litters as well as decomposer abundance and activity, leading to considerable mixing effects on decomposition^[10,19]^. Although litter quality may affect decomposition dynamics^[23]^, the mechanism responsible for the mixing effect on nutrient cycling and decomposer communities still remains unclear.

The composition of the microbial community determines the rate of litter decomposition^[24,25]^. Generally, the composition of microbes shifts from being dominated by fungi at the early stages of decomposition to being dominated by bacteria at the later stages^[26]^. These changes can be attributed to the different structures and functions of the microbial communities^[27,28]^. In terrestrial ecosystems, fungi outperform bacteria in the utilization of more complex and various carbon sources, while bacteria are often superior to fungi in using more labile carbon sources^[29]^. However, a recent study has indicated that diverse bacteria and fungi coexist and interact throughout decomposition^[30]^. Bacteria not only support fungal decomposers by supplying electrons or essential micronutrients but also establish themselves at the soil-litter interface during the breakdown of complex macromolecules by extracellular fungal enzymes^[31]^. These findings imply that bacterial communities may serve as the primary drivers of litter decomposition by modulating patterns of mass loss and contributing to nutrient cycles^[32]^.

In mixed forests, litter mixtures may favor bacterial communities, which depends on the respective biological functions^[33]^, given that the trophic complexity of a decomposer community is crucial for litter decomposition^[34]^. Litter mixtures not only increase the complementary resource utilization by decomposer communities^[19]^, but also provide diverse substrates and niches for microorganisms^[35]^, which can accelerate the litter decomposition rate^[15,36,37]^. Furthermore, microorganisms secrete extracellular enzymes to decompose substrates into smaller compounds for plant nourishment and development^[38]^. The transfer of nutrients from superior to inferior quality litter can occur through either active microbial transfer or passive leaching, as postulated by the nutrient transfer theory^[19]^. Therefore, knowledge on the effects of litter mixtures can contribute to a better understanding of nutrient cycling and feedback mechanisms that regulate species diversity^[39]^. To date, however, few studies have examined the microbial community in terms of the litter decomposition process^[40]^. Although an earlier report has indicated that litter diversity accelerates litter decomposition by increasing the abundance of microorganisms and detritivores^[10]^, empirical studies are rare because of limitations in measurement techniques^[20]^. As a result, how litter mixtures drive microbial community structure and composition remains unclear.

To clarify the effects of litter mixtures on decomposition, we quantified the effects of leaf litter mixtures on litter mass, nutrient loss, and bacterial community structure through a litter-bag experiment in Mount Tai, East China. Our hypotheses were as follows: (1) Litter mixtures can show non-additive effects on litter mass loss due to greater chemical dissimilarity among litters; (2) Decomposition of litter mixtures significantly increases nutrient retention of specific litters, because according to the nutrient transfer theory, nutrients from higher quality litter species are generally transferred to lower quality litter species; and (3) litter mixtures significantly affect the structure and composition of bacterial communities compared with litter monocultures, and the change in litter decomposition rates are due to litter species diversity increases complementary among different decomposers.

## Materials and methods

### Study site

An experiment focusing on leaf litter decomposition was carried out at the Yaoxiang Forest Ecosystem National Positioning Observation Research Station located in Mount Tai in East China. The study area encompassed a total area of 1210.2 hectares, with geographic coordinates of 117°10'E and 36°17'N. This region has a warm, temperate continental monsoon climate, characterized by an annual average temperature of 10.8 °C. The maximum temperature recorded is 34 °C, while the minimum temperature recorded is −24 °C. The average annual precipitation is 950 mm. The soil is typical of mountain brown terrain, with the soil depth ranging from 15 to 90 cm. The forest vegetation mostly consists of planted stands, which were established in the 1950s. The main tree species include *Robinia pseudoacacia*, *Quercus acutissima*, *Pinus tabulaeformis*, and *Pinus densiflora* after decades of reforestation efforts. These species always occur in monospecific stands, with low biodiversity, weak stability, and high vulnerability to pests and diseases, which are unfavorable for ecosystem energy flow and nutrient cycling^[41]^.

### Experimental design

Four litter species, *R. pseudoacacia*, *Q. acutissima*, *P. densiflora*, and *P. tabuliformis*, were collected from four monospecific plantations using litter boxes in the study area in September 2015. The leaf litter was air-dried to constant mass, and a portion of each air-dried litter was oven-dried (65 °C, 48 h) to measure the air- and oven-dried mass ratios. The decomposition experiment was conducted in July 2016 as follows: i) one treatment contained a litter monoculture of four species; 6 g of air-dried leaf litter per species were placed in nylon mesh bags (25 cm × 25 cm, 1-mm mesh size), and ii) the other treatment contained a mixture of two species in all possible pair-wise combinations from four species in equal proportions (six types in total), and each nylon mesh bag (25 cm × 25 cm, 1-mm mesh size) contained two small bags (15 cm × 10 cm, 3 g of air-dried litter per bag) to easily distinguish the litter mixtures. Thus, there were 10 types of litter bags (four monocultures + six mixtures), and each litter type had six replicates. To avoid the effect of home-field advantage on decomposition^[42]^, the litter bags were placed in an area devoid of forests of the Mount Tai Forest Ecosystem Observation and Research Station (36°20′3″N,117°7′11″E). The information, study site climate, and initial litter properties are shown in Supplemental Table S1, Supplemental Fig. S1, and Supplemental Table S2, respectively.

We adopted a randomized, complete block design experiment with six blocks (10 cm × 10 m) that were separated 5 m from each other with similar environmental conditions. Each block included 10 litter bag types, which were pinned to the ground surface.

Litter bags were retrieved in July 2017 after one year of decomposition. Three replicates of each litter type were stored in cryotubes in a liquid nitrogen tank and transferred to the lab for determining the bacterial community composition and structure. The other three replicates were dried at 65 °C for 48 h and weighed after removing adhering soil particles and living plants. Samples were ground for chemical analyses.

The concentrations of C and nitrogen (N) were determined with an elemental analyzer (Costech ECS4010, Costech Analytical Technologies, Valencia CA, Italy), and that of phosphorus (P) by the Mo-Sb antispetrophotography method^[43]^. The lignin concentration was determined by the acid detergent lignin method^[44]^.

### Bacterial DNA extraction and 16S rDNA amplification

The CTAB method^[45]^ was used to extract genomic DNA from the samples, and DNA concentration and purity were assessed by spectroscopy and 1% agarose gel electrophoresis. DNA was diluted to 1 ng/μL with sterile water, and thirty samples served as templates for high throughput sequencing (HTS) analysis, which was conducted by Novogene (Tianjin, China).

To amplify the 16S rDNA genes of distinct regions (16SV4-V5), we employed specific primers [515F (5′-GTGCCAGCMGCCGCGGTAA-3′) and 907R (5′-CCGTCAATTCCTTTGAGTTT-3′)], along with the addition of barcode sequences. All polymerase chain reactions (PCR) were performed using the Phusin High-Fidelity PCR Master Mix (New England Biolabs, Ipswich, MA, USA). Subsequently, PCR products were combined with an equal volume of 1× loading buffer (containing SYB green) and subjected to 2% agarose gel electrophoresis. Samples displaying a clear, prominent band of 400–450 bp were selected for further study. To ensure a balanced representation, the PCR products were combined according to equal density ratios. Combined PCR products were purified using the Qiagen Gel Extraction Kit (Qiagen, Germany). For library construction, the TruSeq DNA PCR-Free Sample Preparation Kit (Illumina, San Diego, CA, USA) was used according to the manufacturerʼs instructions, in addition to the incorporation of index codes. The quality of the library was evaluated using the Qubit@2.0 fluorometer (Thermo Fisher Scientific, Waltham, MA, USA) and the Agilent Bioanalyzer 2,100 instrument (Santa Clara, CA, USA). Finally, the library was sequenced on the Illumina HiSeq 2500 platform to generate 250-bp paired-end reads.

### Statistical analysis

The percentage of litter mass remaining (percentage of initial mass) (D) and the nutrient retention rate (R) were calculated as follows^[16]^:

1 $ D({\%}) = M_{\rm t}/M_{0}\times100{\%} $

2 $ R({\%}) = (C_{\rm t}\times M_{\rm t})/(C_{0}\times M_{0})\times 100{\%} $

Where *M*_0_ and *M*_t_ are the oven-dried weights of leaf litter before and after decomposition, respectively; *C*_0_ is the initial concentration of C, N, P, or lignin; and *C*_t_ is the concentration of these elements as a percentage of litter mass at each sampling event.

The predictive mass of the litter mixtures was calculated as follows^[46]^:

3 $ {\mathrm{Predictive \;value}} = [M_{1}/(M_{1}+M_{2})]\times R_{1}+[M_{2}/(M_{1}+M_{2})]\times R_{2} $

Where *R*_1_ and *R*_2_ are the percentages of the mass remaining in the single species litter-bag of species 1 and 2, respectively, and *M*_1_ and *M*_2_ are the estimated initial litter dry masses of these species in the mixture.

Sequencing reads from the dataset were trimmed, quality-controlled, and aligned. Operational taxonomic units (OTUs) were clustered at 97% identity using Uparse (v7.0.1001, http://drive5.com/uparse/). Taxonomic classification was conducted using RDP classifier (v2.2, http://sourceforge.net/projects/rdp-classifier/). Alpha diversity was used to analyze the diversity of bacterial species for each sample based on three different diversity indices, Chao 1, Shannon, and ACE, which were calculated with QIIME (v1.9.1) and displayed using R software (v2.15.3, https://cran.r-project.org/).

To clarify the mixing effects, significance of the differences between the observed and predicted decomposition values was assessed by a single sample *t*-test for each mixture treatment. A non-additive effect was defined as a significant difference between the observed and predicted decomposition values; otherwise the effect was considered to be additive. Nonmetric multidimensional scaling (NMDS) was used to examine the differences in bacterial community structure between the litter-bag types using Bray–Curtis distances^[47]^. Analysis of similarities (ANOSIM) was used to examine the significance of bacterial community structure on litter types^[48]^. Pearson's correlation analysis was conducted to explore relationships between the litter nutrient retention and alpha diversity. Spearman's correlation analysis and redundancy analysis (RDA) were applied to determine the main factors driving litter decomposition and bacterial community structure^[24]^. Correlation analysis and one-way analysis of variance (ANOVA) were conducted in SPSS 26.0 (IBM Armonk, NY, USA). NMDS and RDA were performed using R (v2.15.3) to examine relationships between the bacterial community structure and initial nutrient concentration of litter.

## Results

### Differences in litter mass remaining of mixtures

**Figure 1:**
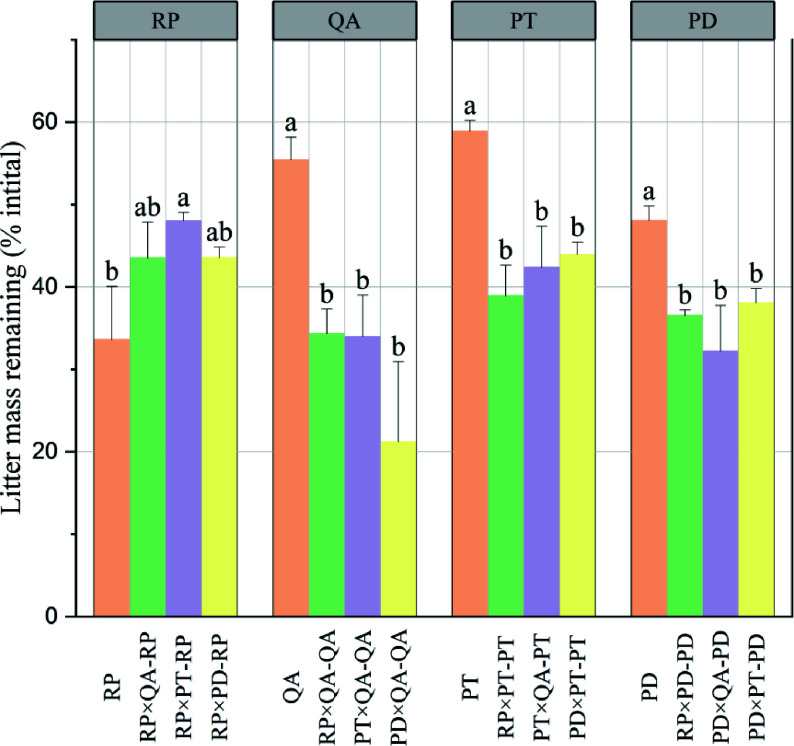
Litter mass remaining in decomposing monocultures and mixtures. × represents mixed decomposition. A×B-A and A×B-B represent decomposition characteristics of A and B in mixed decomposition, respectively. Mean ± standard error (SE), n = 3. Error bars represent SE. Different lowercase letters indicate significant differences (*p* < 0.05) among different types of litter-bag. RP, *Robinia pseucdoacacia*; QA, *Quercus acutissima*; PT, *Pinus tabulaeformis*; and PD, *Pinus densiflora*.

**Figure 2:**
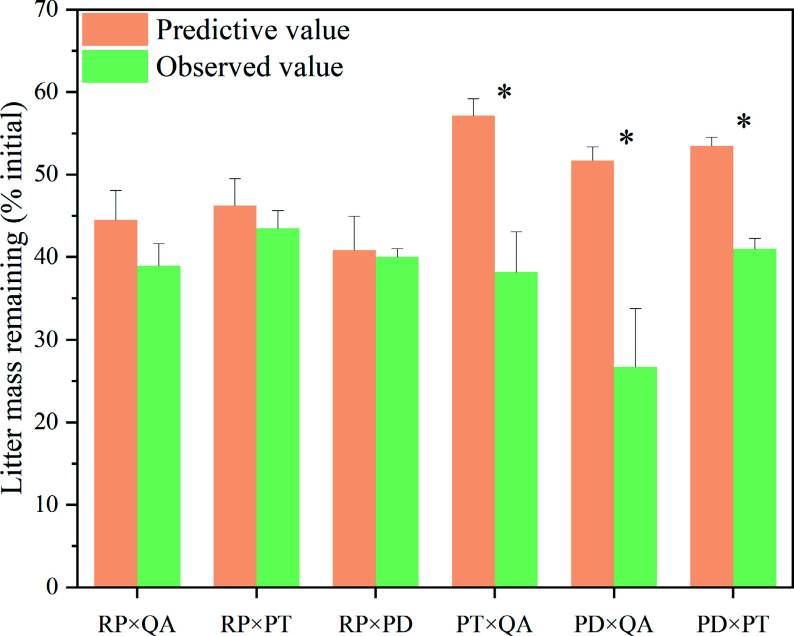
Observed and predicted litter mass remaining for litter mixtures. × represents mixed decomposition. Mean ± SE, n = 3. * indicates significant differences (*p* < 0.05) between predictive and observed values among different types of litter-bag. RP, *Robinia pseucdoacacia*; QA, *Quercus acutissima*; PT, *Pinus tabulaeformis*; and PD, *Pinus densiflora*.

After one year, except for *R. pseudoacacia*, the litter mass remaining in monocultures (33.61%–8.90%) was significantly higher than that in mixtures (21.21%–48.05%) with no significant difference among the three mixtures (Fig. 1). For *R. pseudoacacia*, there was no difference between the monocultures and mixtures, except for the *P. tabuliformis* mixture (Fig. 1). The litter mass remaining for mixtures without *R. pseudoacacia* [*P. tabuliformis* × *Q. acutissima* (38.17%), *P. densiflora* × *Q. acutissima* (26.72%), and *P. densiflora* × *P. tabuliformis* (41.00%)] was significantly lower than the predicted values (*p* < 0.05) (57.15%, 51.73%, and 53.48, respectively), suggesting non-additive synergistic effects. On the other hand, the litter mass remaining for the three mixtures with *R. pseudoacacia* had no significant difference in the predicted values (*p* > 0.05), suggesting additive effects (Fig. 2).

### Differential litter nutrient retention among different litter types

**Figure 3:**
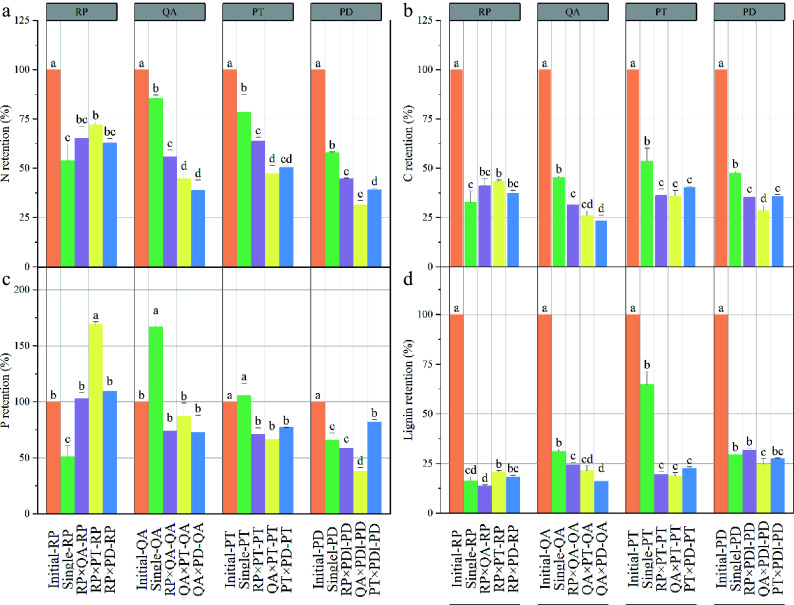
Nutrient retention rates of litter in monocultures and mixtures after one year's decomposition. × represents mixed decomposition. Error bars represent standard errors. Different lowercase letters above the bars indicate significant differences (*p* < 0.05) between the different litter-bag types. A, B, C, and D represent N, C, P, and lignin retention, respectively. RP*, Robinia pseucdoacacia*; QA, *Quercus acutissima*; PT, *Pinus tabulaeformis*; and PD, *Pinus densiflora*.

Litter decomposition significantly decreased the retention rates of litter C and N in both monocultures and mixtures (Fig. 3a, b). The P retention rate showed a similar decreasing trend in two conifers (*P. densiflora* and *P. tabuliformis*) but a dissimilar trend in two broadleaves species (*R. pseudoacacia* and *Q. acutissima*). For *R. pseudoacacia*, the P retention rate increased in the *P. tabuliformis* mixtures and decreased in the monocultures. For *Q.* *acutissima*, the P retention rate increased in the monocultures (Fig. 3c). Furthermore, the nutrient retention rate in monocultures was higher than that in mixtures, except for *R. pseudoacacia* (Fig. 3). For *R. pseudoacacia*, the retention rate of litter C, N, and lignin in monocultures were similar with those in mixtures, except for the *P. tabuliformis* mixture (Fig. 3a, b, d), and it showed a similar trend with the litter mass remaining (Fig. 1). Compared with the litter monocultures, the decomposition of litter mixtures significantly increased the P retention rate of *R. pseudoacacia*, irrespective of the mixture type (Fig. 3c).

### Composition and diversity of bacterial communities

**Figure 4:**
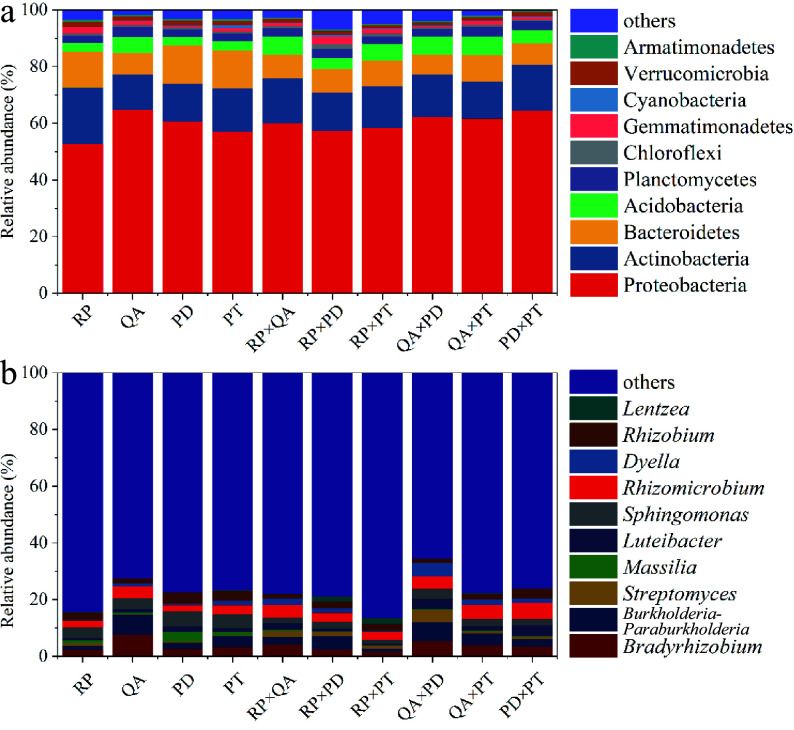
Composition of the 10 most abundant taxonomic groups according to the mean relative abundances of bacterial assemblages, (a) at the phylum level and (b) at the genus level. × represents mixed decomposition. RP, *Robinia pseucdoacacia*; QA, *Quercus acutissima*; PT, *Pinus tabulaeformis*; and PD, *Pinus densiflora*.

In total, 52,953 effective tags were used for analyzing the composition and diversity of bacterial communities, and they were clustered into 2,275 OTUs at a 97% similarity level. The sequences were assigned to 36 phyla and 447 genera (Fig. 4). Proteobacteria and Actinobacteria were the main phyla across all samples after decomposition for one year, accounting for 57.5%–64.8% and 12.5%–17.3% of the total valid reads, respectively (Fig. 4a). At the genus level, the groups with average relative abundance higher than 2% were *Bradyrhizobium* (3.8%), *Burkholderia*-*Paraburkholderia* (3.6%), *Sphingomonas* (3.2%), *Rhizomicrobium* (3.6%), and *Rhizobium* (2.5%) (Fig. 4b).

**Figure 5:**
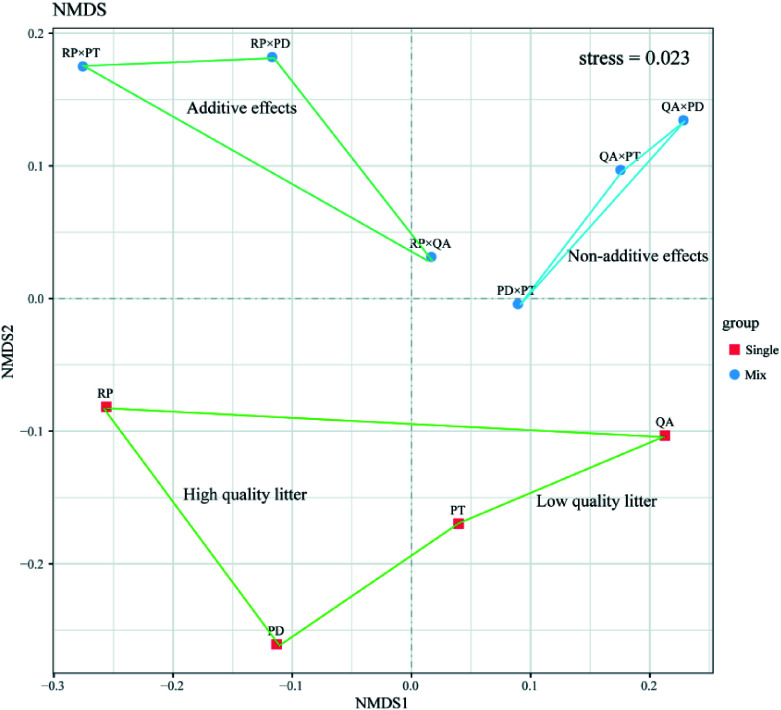
Bacterial community structure in leaf litter samples decomposed for one year using Bray–Curtis distances. Squares and circles show bacterial community structures of litter monocultures and mixtures, respectively. The stress value was 0.023. RP, *Robinia pseucdoacacia*; QA, *Quercus acutissima*; PT, *Pinus tabulaeformis*; and PD, *Pinus densiflora*.

For the monocultures, the bacterial species richness (Chao1 and Ace) of the two broadleaves (*R. pseudoacacia* and *Q. acutissima*) was higher than that of the two conifers (*P. densiflora* and *P. tabuliformis*). The Shannon index of the *Q. acutissima* litter was significantly lower than that of the other three litter types (*p* < 0.05) (Supplemental Table 3). For the mixtures, the bacterial α-diversity of the three mixtures with *P. densiflora* (*P. densiflora* × *R. pseudoacacia*, *P. densiflora* × *Q. acutissima*, and *P. densiflora* × *P. tabuliformis*) was lower than that of the other mixtures. However, the bacterial α-diversity of the mixtures of broad-leaved tree species (*R. pseudoacacia* or *Q. acutissima*) with *P. tabuliformis* was significantly higher than that of the other mixtures, especially for *R. pseudoacacia* × *P. tabuliformis* (Supplemental Table 4). Both NMDS (Fig. 5) and ANOSIM revealed that mixtures with *R. pseudoacacia* (additive effects), mixtures without *R. pseudoacacia* (non-additive effects), and those in monocultures had distinct bacterial community structures (ANOSIM *R* = 0.369, *p* = 0.022).

### Linking litter mass remaining to bacterial community and initial litter properties

**Figure 6:**
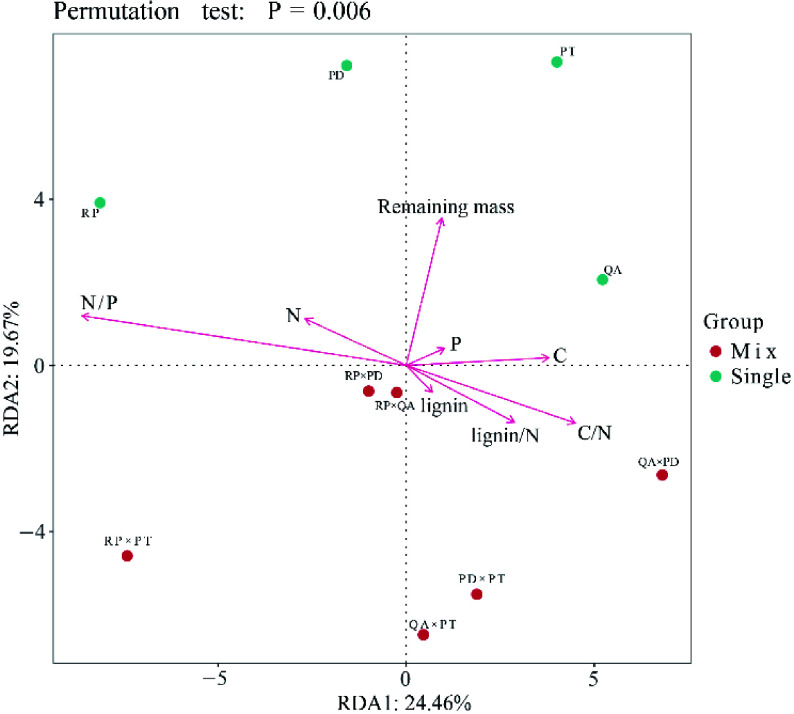
Redundancy analysis (RDA) of bacterial community structure, litter mass remaining, and initial litter properties. Red and blue circles represent bacterial community structure of litter monocultures and mixtures, respectively. Red lines represent the litter mass remaining and initial litter properties. RP, *Robinia pseucdoacacia*; QA, *Quercus acutissima*; PT, *Pinus tabulaeformis*; and PD, *Pinus densiflora*.

The relative abundance of the bacterial community was mainly correlated with the initial litter C/N, N/P and lignin/N ratios (Supplemental Tables 5 & 6), especially at the genus level. The relative abundance was positively associated with the initial litter C/N and lignin/N ratios and negatively associated with the N/P ratio (*p* < 0.05, Supplemental Table 6). Bacterial community structure in both litter monocultures and mixtures was driven by initial litter properties (Fig. 6). In monocultures, the bacterial community structure mainly depended on initial C, N, and P concentrations, and there was great difference between high quality litters (N concentrations) and low quality litters (C and P concentrations). In mixtures, the bacterial community structure in mixtures with *R. pseudoacacia* (additive effects) was mainly determined by the initial N/P ratio, and mixtures without *R. pseudoacacia* (non-additive effects) were mainly determined by C/N and lignin/N ratios (Fig. 6, Supplemental Table 7).

## Discussion

### Non-additive and additive effects occur concurrently in decomposition of mixed litters

We reveal that litter decomposition is influenced by the specific characteristics of each litter species within a mixture. The different litter species included in the mixtures exhibited varying responses to decomposition^[49]^. As hypothesized, the addition or exclusion of *R. pseudoacacia* in litter mixtures resulted in additive or non-additive synergistic effects on litter decomposition, respectively. These outcomes are consistent with previous research, which indicated that litter types influence litter mixing effects^[19,50]^. The mixture of three native species (*Q. acutissima*, *P. densiflora*, and *P. tabuliformis*) showed synergistic effects on litter decomposition. This observation may be attributed to the long-term adaptation of local decomposer communities, which favor synergistic effects that originated from the combination of different litter types^[49]^. Our study provides further evidence that the composition of species greatly impacts the intensity of non-additive effects in temperate plantations.

Great dissimilarities in litter compounds can produce synergistic effects on the decomposition of mixed litters. For instance, N is transferred from the N-rich to the N-poor litter to consequently enhance the microbial decomposition of poor-quality litter^[19,51]^. Thus, litter mixtures with higher N concentrations are expected to yield synergistic effects on litter decomposition. However, we found that litter mixtures with high-N concentrations containing *R. pseudoacacia* exerted additive effects (Fig. 2), which is inconsistent with previous findings^[17,47]^. One explanation could be that plant litter containing specific compounds may inhibit microbial activities and produce antagonistic effects on adjacent component litters^[52]^. The low decomposition rate of litter mixtures containing *R. pseudoacacia* can be detrimental to nutrient release^[6,53]^. Furthermore, *R. pseudoacacia*, an invasive N-fixing species, does not require much surrounding nutrient^[54,55]^. Therefore, *R. pseudoacacia* can reduce the competitiveness of native plants by inhibiting the litter decomposition rate during the decomposition of mixed litter. From this perspective, the litter mixing effects may explain the phenomenon of biological invasion.

### Effects of mixed litter decomposition on nutrient retention

The decomposition of mixed litter significantly changed the nutrient retention rate of specific litters in the mixtures, supporting our second hypothesis that decomposition of litter mixtures significantly increases nutrient retention of specific litters. The decomposition rate of *R. pseudoacacia* in the litter mixtures showed no significant difference from that in the monocultures, and the nutrient retention rate, especially that of P, increased mostly in the mixtures, consistent with previous findings^[6,51]^. Invasion of *R. pseudoacacia* into grassland ecosystems can have a significant impact on temperature and light conditions in the understory^[56]^. This invasion has been shown to decrease the abundance and richness of microarthropods and nematodes, as well as plant diversity^[54]^. It may also be related to the presence of allelochemicals such as acacetin and quercetin in *R. pseudoacacia* litter^[57]^. These findings suggest that nutrient cycling in mixed plantations can be negatively affected by *R. pseudoacacia* litter. These findings also highlight the importance of understanding how plants acquire nutrients, as this may play a key role in the decomposition of mixed litter in temperate forest plantations. Therefore, it is important to decrease *R. pseudoacacia* expansion to maintain temperate forest ecosystems.

Compared with monocultures, the presence of *Q. acutissima*, *P. densiflora*, and *P. tabuliformis* in mixtures significantly decreased the litter mass remaining and the nutrient retention rate, suggesting a strong synergistic effect when these species are combined. A previous study has shown that the presence of invasive species in litter can increase the rates of decomposition and release of C, N, and P from the litter of native species^[58]^. The synergistic effects observed in litter mixtures without *R. pseudoacacia* may be attributed to the nutrient transfer theory. According to this theory, nutrients are transferred from higher quality litter to lower quality litter through either active microbial transfer or passive leaching^[19]^. The other reason may be the home-field advantage of local microclimatic conditions and decomposer communities for native rather than invasive species^[49]^. Therefore, mixed litters comprised of native species promote its own decomposition as well as that of other litters, consequently accelerating nutrient cycling. These findings may explain why mixed plantation systems are relatively stable. These synergistic effects suggest that species combinations can be applied to mixed plantations.

### Structure and composition of the bacterial community significantly affects the mixing effects through nutrient links

Litter monocultures and mixtures distinctly drove the litter bacterial community structure due to differences in the initial litter properties. Bacterial community structure was determined by initial litter C, N, and P concentrations of monocultures, and C/N, N/P, and lignin/N ratios of mixtures. These findings suggest that chemical elements, as fundamental resources for decomposition in the food web, are indispensable for microbial reproduction^[59,60]^. Chemical element ratios in litter materials shape the structure of decomposer communities^[61]^. Microbes have specific nutrient requirements for their metabolic processes related to energy and growth, which can alter decomposition processes. Our findings demonstrated that the bacterial diversity (α-diversity) of the broadleaved litter was considerably greater than that of the coniferous litter. The broadleaved litter, which is characterized by its high-quality composition and rapid decomposition, releases significant amounts of basic cations such as Ca and Mg that may contribute to maintaining a lower soil acidification, which changes the bacterial community composition and activity^[37,62,63]^. In addition, bacterial α-diversity was generally higher in litter mixtures than in litter monocultures because mixtures can combine complementary resources to meet the requirements of various bacteria^[64]^.

Changes in bacterial community composition may also be driven by litter matrix nutrient availability^[27]^. Greater nitrogen (N) and lower lignin contents (indicated by a lower lignin/N ratio) result in the higher availability of energy and nutrients, along with reduced resistance to decomposition, thereby accelerating microbial activities^[65]^. Our results showed that Proteobacteria and Actinobacteria were the main phyla involved in litter decomposition, accounting for 57.5%–64.8% and 12.5%–17.3% of the total valid reads, respectively. Furthermore, *R. pseudoacacia* in monocultures and mixtures had a lower abundance of Proteobacteria than other treatments. The relative abundance of Proteobacteria exhibited a positive correlation with the initial carbon-to-nitrogen (C/N) ratio and a negative correlation with the initial nitrogen-to-phosphorus (N/P) ratio because Proteobacteria is the dominant phylum in litter decomposition processes and conducive to C and N cyclings^[66]^. Furthermore, Actinobacteria produces multiple degradation enzymes^[67]^. Although the abundance of Actinobacteria was higher in the *R. pseudoacacia* litter, its abundance was lower in litter mixtures with *R. pseudoacacia*. Therefore, the decline in the population of Proteobacteria and Actinobacteria when combined with *R. pseudoacacia* could be the primary reason for the additional impact on the decomposition of mixed litter.

At the genus level, microbes are sensitive to initial litter properties^[27]^. Our results showed that *Bradyrhizobium* was positively correlated with the C/N ratio, which is consistent with a report by Janssens et al.^[68]^, who showed that a lower *Bradyrhizobium* abundance significantly reduced N fixation and increased decomposition. *Burkholderia* is negatively correlated with the N/P ratio because *Burkholderia* is a P solubilizer. Among the various microorganisms, only *Sphingomonas* exhibits a positive correlation with the remaining litter mass, as it can regulate C and N metabolism and degrade aromatic organic compounds^[69]^. During the entire process, litter decomposition selectively stimulates and increases the abundance of *Sphingomonas*^[70]^. Therefore, the relatively lower abundance of *Sphingomonas* in litter mixtures compared to littler monocultures may explain the non-additive effects observed in the decomposition of mixed litter. However, because of the short decomposition time, there is still an urgent need for more observational data to support the findings of this study.

## Conclusions

Our study provides direct field evidence that the decomposition of mixed litter is influenced by the type of litter mixture, which is mediated by the interactive modulation of litter properties and the composition of bacterial communities. Litter mixtures without *Robinia pseudoacacia* showed non-additive synergistic effects on litter decomposition, whereas litter mixtures with *R. pseudoacacia* exerted additive effects. These results indicate that nutrient release in mixtures was faster than that in monocultures, except for those with *R. pseudoacacia*. *R. pseudoacacia* may slow down ecosystem nutrient cycling, thus facilitating its invasion. Litter mixtures significantly modified the structure and composition of the bacterial community through nutritional links with litter traits. These findings have expanded our understanding of the mixing effects and microbial mechanisms underlying the acceleration of litter decomposition in mixed plantations compared with monoculture plantations, which indicate that the non-additive effects from mixed plantations are crucial for forest restoration and ecosystem health.

## SUPPLEMENTARY DATA

Supplementary data to this article can be found online.

## Data Availability

The datasets generated during and/or analyzed during the current study are available from the corresponding author on reasonable request.
