# Supplementary material for: Leaf litter mixtures alter decomposition rate, nutrient retention, and bacterial community composition in a temperate forest
Source: For Res (Fayettev). 2023 Sep 27;3:22. doi: 10.48130/FR-2023-0022 (PMC11524288; doi:10.48130/FR-2023-0022)
Supplement: Supplementary file 1 — Supplementary data to this article can be found online. [file FR-2023-0022-S1.zip › 10.48130_FR-2023-0022-Suppl-TableS1.pdf]

Tab. S1 Information of forests areas

Elevation (m)	Slope degree (°)	Slope aspect	Soil layer depth (cm)	pH	C (%)	N (%)	Soil organic carbon (g/kg)
730	23	South	26.08	5.00	2.13	0.15	9.32
