# Supplementary material for: Leaf litter mixtures alter decomposition rate, nutrient retention, and bacterial community composition in a temperate forest
Source: For Res (Fayettev). 2023 Sep 27;3:22. doi: 10.48130/FR-2023-0022 (PMC11524288; doi:10.48130/FR-2023-0022)
Supplement: Supplementary file 1 — Supplementary data to this article can be found online. [file FR-2023-0022-S1.zip › 10.48130_FR-2023-0022-Suppl-TableS2.pdf]

Tab.S2 Initial litter properties of four leaf litter types.

	C%	N/%	P%	Lignin/%	C/N	N/P	Lignin/N
RP	45.57±0.13c	1.86±0.05a	0.41±0.01c	28.84±0.36c	24.58±0.57b	4.55±0.27a	15.55±0.28c
QA	48.23±0.84b	1.21±0.03b	0.43±0.01c	33.63±0.57b	39.91±1.03a	2.82±0.08c	27.81±0.24a
PD	50.64±0.15a	2.02±0.01a	0.59±0.01a	22.83±0.54d	25.10±0.16b	3.42±0.08b	11.32±0.33d
PT	50.34±0.51a	1.90±0.08a	0.54±0.01b	37.21±0.61a	26.64±0.90b	3.50±0.16b	19.70±0.85b

Note: Values are means ± SE. RP*, Robinia pseucdoacacia*; QA, *Quercus acutissima*; PT, *Pinus tabulaeformis*; PD, *Pinus densiflora*. C, total carbon; N, total nitrogen; P, total phosphorus; C/N, carbon:nitrogen ratios; N/P, nitrogen:phosphorus ratios; Lignin:N, Lignin/nitrogen ratios. Different lowercase letters represent significant differences among four leaf litter types.
