# Supplementary material for: Leaf litter mixtures alter decomposition rate, nutrient retention, and bacterial community composition in a temperate forest
Source: For Res (Fayettev). 2023 Sep 27;3:22. doi: 10.48130/FR-2023-0022 (PMC11524288; doi:10.48130/FR-2023-0022)
Supplement: Supplementary file 1 — Supplementary data to this article can be found online. [file FR-2023-0022-S1.zip › 10.48130_FR-2023-0022-Suppl-TableS3.pdf]

Tab. S3 Bacterial α-diversity in the monoculture litter after one-year decomposition.

Litterbag types	Chao1	Ace	Shannon
RP	2729.5±92.5a	2731.0±109.0a	9.13±0.11a
QA	2672.9±19.1a	2706.4±65.9a	8.38±0.07c
PD	2221.5±86.4b	2282.3±98.8b	8.97±0.52ab
PT	2275.8±88.7b	2311.5±60.5b	8.78±0.03b

Note: Values are means ± SE (n=3). RP*, Robinia pseucdoacacia*; QA, *Quercus acutissima*; PT, *Pinus tabulaeformis*; PD, *Pinus densiflora*. Different lowercase letters represent significant differences among four leaf litter types.
