# Supplementary material for: Leaf litter mixtures alter decomposition rate, nutrient retention, and bacterial community composition in a temperate forest
Source: For Res (Fayettev). 2023 Sep 27;3:22. doi: 10.48130/FR-2023-0022 (PMC11524288; doi:10.48130/FR-2023-0022)
Supplement: Supplementary file 1 — Supplementary data to this article can be found online. [file FR-2023-0022-S1.zip › 10.48130_FR-2023-0022-Suppl-TableS4.pdf]

Tab. S4 Bacterial α-diversity in mixtures after one year's decomposition

Litterbag types	Chao1	Ace	Shannon
RP×QA	3109.5±251.3bc	2651.4±89.5abc	8.88±0.19a
RP×PD	2583.9±159.7c	2329.0±94.2bc	8.61±0.24ab
RP×PT	3879.8±150.9a	3237.6±208a	8.87±0.29a
QA×PD	2597.2±312.1c	2189.6±387.2c	7.88±0.62b
QA×PT	3365.7±158.8ab	2875.6±129.8ab	8.83±0.37a
PD×PT	3129.8±192.0bc	2603.2±76.1abc	8.53±0.13ab

Note: Values are means ±SE (n=3). RP*, Robinia pseucdoacacia*; QA, *Quercus acutissima*; PT, *Pinus tabulaeformis*; PD, *Pinus densiflora*. "×" represents mixing decomposition. Different lowercase letters represent significant differences among four leaf litter types.
