# Supplementary material for: Leaf litter mixtures alter decomposition rate, nutrient retention, and bacterial community composition in a temperate forest
Source: For Res (Fayettev). 2023 Sep 27;3:22. doi: 10.48130/FR-2023-0022 (PMC11524288; doi:10.48130/FR-2023-0022)
Supplement: Supplementary file 1 — Supplementary data to this article can be found online. [file FR-2023-0022-S1.zip › 10.48130_FR-2023-0022-Suppl-TableS5.pdf]

Tab. S5 Spearman correlations of relative abundance of phylum with initial litter properties and with litter mass remaining.

	N%	C/%	C/N	P	Lignin%	N/P	Lignin/N	Mass remaining
Proteobacteria	-0.304	0.149	0.386*	0.120	0.097	-0.479**	0.297	-0.141
Actinobacteria	-0.015	-0.168	-0.017	-0.129	-0.152	0.247	-0.128	-0.128
Bacteroidetes	0.184	0.065	-0.220	0.085	-0.050	0.109	-0.207	0.156
Acidobacteria	-0.265	-0.022	0.289	-0.201	0.247	-0.155	0.390*	-0.123
Planctomycetes	-0.151	0.022	0.178	-0.139	0.163	-0.163	0.267	0.196
Chloroflexi	0.165	-0.361	-0.363*	-0.271	-0.052	0.477**	-0.201	-0.043
Gemmatimonadetes	0.023	-0.510**	-0.252	-0.373*	0.042	0.445 *	-0.100	-0.064
Cyanobacteria	0.039	0.087	-0.032	-0.122	0.098	0.024	0.114	0.035
Verrucomicrobia	0.465**	0.165	-0.437*	0.150	-0.230	0.197	-0.383*	0.398*
Armatimonadetes	0.193	0.039	-0.180	-0.001	0.159	0.101	-0.074	0.404 *

* represents $p<0.05$; ** represents $p<0.01$.
