# Supplementary material for: Leaf litter mixtures alter decomposition rate, nutrient retention, and bacterial community composition in a temperate forest
Source: For Res (Fayettev). 2023 Sep 27;3:22. doi: 10.48130/FR-2023-0022 (PMC11524288; doi:10.48130/FR-2023-0022)
Supplement: Supplementary file 1 — Supplementary data to this article can be found online. [file FR-2023-0022-S1.zip › 10.48130_FR-2023-0022-Suppl-TableS6.pdf]

Tab. S6 Spearman correlations of relative abundance of genus with initial litter properties and with litter mass remaining.

	N%	C/%	C/N	P	Lignin%	N/P	Lignin/N	Mass remaining
Bradyrhizobium	-0.385*	0.145	0.484**	-0.093	0.190	-0.494**	0.465**	0.005
Burkholderia.Paraburkholderia	-0.395 *	0.206	0.501**	-0.032	0.105	-0.539**	0.424*	-0.034
Streptomyces	0.171	-0.373*	-0.316	-0.262	-0.237	0.541**	-0.249	-0.314
Massilia	-0.069	-0.096	0.025	-0.087	-0.004	-0.032	-0.029	0.319
Luteibacter	-0.135	0.424*	0.315	0.363*	-0.057	-0.525**	0.151	0.105
Sphingomonas	0.004	0.245	0.083	0.126	-0.067	-0.249	-0.046	0.362*
Dyella	-0.319	0.267	0.455*	0.139	0.155	-0.447*	0.374*	-0.244
Rhizomicrobium	-0.305	0.088	0.392*	-0.039	0.301	-0.338	0.425*	-0.197
Rhizobium	0.172	0.198	-0.128	0.308	0.009	-0.018	-0.183	0.047
Lentzea	0.197	-0.323	-0.320	-0.126	-0.124	0.491**	-0.305	-0.165

* represents $p<0.05$; ** represents $p<0.01$.
