# Supplementary material for: Leaf litter mixtures alter decomposition rate, nutrient retention, and bacterial community composition in a temperate forest
Source: For Res (Fayettev). 2023 Sep 27;3:22. doi: 10.48130/FR-2023-0022 (PMC11524288; doi:10.48130/FR-2023-0022)
Supplement: Supplementary file 1 — Supplementary data to this article can be found online. [file FR-2023-0022-S1.zip › 10.48130_FR-2023-0022-Suppl-TableS7.pdf]

Tab. S7 RDA results of bacterial community structure, litter mass remaining and initial litter properties.

Litterbag types	RDA1	RDA2	Initial litter properties	RDA1	RDA2	R^2	P
RP	-8.14	3.91	N	-0.92	0.38	0.25	0.37
QA	5.22	2.06	C	1.00	0.05	0.32	0.24
PD	-1.58	7.21	C/N	0.96	-0.29	0.40	0.17
PT	4.01	7.30	P	0.93	0.37	0.09	0.68
RP×QA	-0.25	-0.66	lignin	0.73	-0.68	0.08	0.73
RP×PD	-1.00	-0.62	N/P	-0.99	0.14	0.74	0.01
RP×PT	-7.42	-4.58	Lignin/N	0.90	-0.43	0.27	0.33
QA×PD	6.81	-2.63	Mass remaining	0.26	0.97	0.31	0.28
QA×PT	0.46	-6.48					
PD×PT	1.88	-5.51					
